# Early Detection of Bacteraemia Using Ten Clinical Variables with an Artificial Neural Network Approach

**DOI:** 10.3390/jcm8101592

**Published:** 2019-10-02

**Authors:** Kyoung Hwa Lee, Jae June Dong, Su Jin Jeong, Myeong-Hun Chae, Byeong Soo Lee, Hong Jae Kim, Sung Hun Ko, Young Goo Song

**Affiliations:** 1 Division of Infectious Diseases, Department of Internal Medicine, Gangnam Severance Hospital, Yonsei University College of Medicine, 211 Eonju-ro, Gangnam-gu, Seoul 06273, Korea; khlee0309@yuhs.ac; 2 Department of Family Medicine, Gangnam Severance Hospital, Yonsei University College of Medicine, Seoul 06273, Korea; s82tonight@yuhs.ac; 3 Division of Infectious Diseases, Department of Internal Medicine, Severance Hospital, Yonsei University College of Medicine, Seoul 03722, Korea; JSJ@yuhs.ac; 4 Selvas Artificial Intelligence Incorporate, Seoul 08594, Korea; victor.m.chae@selvas.com (M.-H.C.);; 5 Department of Medical Information, Gangnam Severance Hospital, Seoul 06273, Korea; khj1123@yuhs.ac (H.J.K.); ydhun05@yuhs.ac (S.H.K.)

**Keywords:** artificial intelligence, bacteraemia, prediction, artificial neural network, machine learning

## Abstract

An adequate model for predicting bacteraemia has not yet been developed. This study aimed to evaluate the performance of an artificial neural network (ANN)-based prediction model in comparison with previous statistical models. The performance of multi-layer perceptron (MLP), a representative ANN model, was verified via comparison with a non-neural network model. A total of 1260 bacteraemia episodes were identified in 13,402 patients. In MLP with 128 hidden layer nodes, the area under the receiver operating characteristic curve (AUC) of the prediction performance was 0.729 (95% confidence interval [CI]; 0.712–0.728), while in MLP with 256 hidden layer nodes, it was 0.727 (95% CI; 0.713–0.727). In a conventional Bayesian statistical method, the AUC was 0.7. The aforementioned two MLP models exhibited the highest sensitivity (0.810). The ranking of clinical variables was used to describe the influential power of the prediction. Serum alkaline phosphatase was one of the most influential clinical variables, and one-out search was the best ranking method for measuring the influence of the clinical variables. Furthermore, adding variables beyond the 10 top-ranking ones did not significantly affect the prediction of bacteraemia. The ANN model is not inferior to conventional statistical approaches. Bacteraemia can be predicted using only the top 10 clinical variables determined by a ranking method, and the model can be used in clinical practice by applying real-time monitoring.

## 1. Introduction

Evidence-based medicine, which is the basis of modern medicine, involves making medical judgments based on scientific evidence and clinical experience [1]. Therefore, big data analysis is attracting attention owing to the vast amount of information available thanks to recent advances in medicine [2,3,4]. Most analyses using artificial intelligence techniques focus on the diagnosis of chronic diseases, medical imaging, or health care control [4,5,6]. Predictive models of acute infectious diseases are applied in special circumstances, such as in emergency rooms, intensive care units, or for specific infectious diseases [7,8,9,10,11]; however, few models predict bacteraemia prognosis in general medical conditions. By applying machine learning-based algorithms using artificial intelligence techniques to massive amounts of medical data, we attempt to build a real-time monitoring system for the prediction of diseases to support accurate, efficient, and timely clinical decision-making in any situation [12,13].

Acute-phase diseases, especially acute infectious diseases or sepsis, are a major cause of mortality. Early diagnosis and active treatment can improve the outcome in such cases [14]. Various biomarkers such as procalcitonin, presepsin, and CD64 have been studied in early-onset models of acute and severe infectious diseases; however, the application of such biomarkers in clinical practice is insufficient [15,16,17]. Previously, we presented a model for predicting bacteraemia using a Bayesian statistical approach [18], in which bacteraemia was predicted on the basis of the statistical stratification of clinical variables. There was a limitation linked to the presence of structural and unstructured variables in the aforementioned model.

Therefore, it is necessary to develop a model that can predict acute and severe infectious diseases with an unstratified analysis and thus, predict bacteraemia, which is observed in patients suffering from serious infectious diseases. Improving the prediction of bacteraemia for patients in a hospital is a big challenge. We have developed various machine learning prediction models to achieve a better prediction performance. In this study, we aimed to evaluate the performance of an artificial neural network (ANN)-based prediction model in comparison with previous traditional statistical models and use artificial intelligence as a starting point for the analysis and prediction of progress in acute severe infectious diseases.

## 2. Materials and Methods

### 2.1. Definition of Bacteraemia

Patients who presented bacteraemia in more than one blood culture within 24 h were considered to have the same episode. Furthermore, if bacteraemia was present in additional blood cultures from the same patient in the following 24 h, this was considered a new episode [19]. All blood culture episodes meeting these criteria were included in the study. The primary outcome was true bacteraemia, and common contaminants such as *Bacillus* spp., viridans streptococci, and coagulase-negative staphylococci, were excluded from true bacteraemia on the basis of the United States National Healthcare Safety Network system [20,21]. These results of contaminants were regarded as non-bacteraemia. We attempted to compare the probability of direct prediction using the same datasets as those used in the previous Bayesian study [18]. Thus, the true bacteraemia group was analysed in comparison to the non-bacteraemia group.

### 2.2. Study Population

We retrospectively analysed 13,402 patients, above the age of 18, who were admitted to Gangnam Severance Hospital, in Seoul, South Korea, between July 2008 and February 2012 and were subjected to a blood culture test. A total of 22,332 blood culture episodes were collected from these patients. We predicted the probability of bacteraemia risk in the patients who were subjected to the blood culture tests. We excluded 2265 blood culture episodes as more than 80% of the total variables in the data from these tests were lost. 

**Figure 1:**
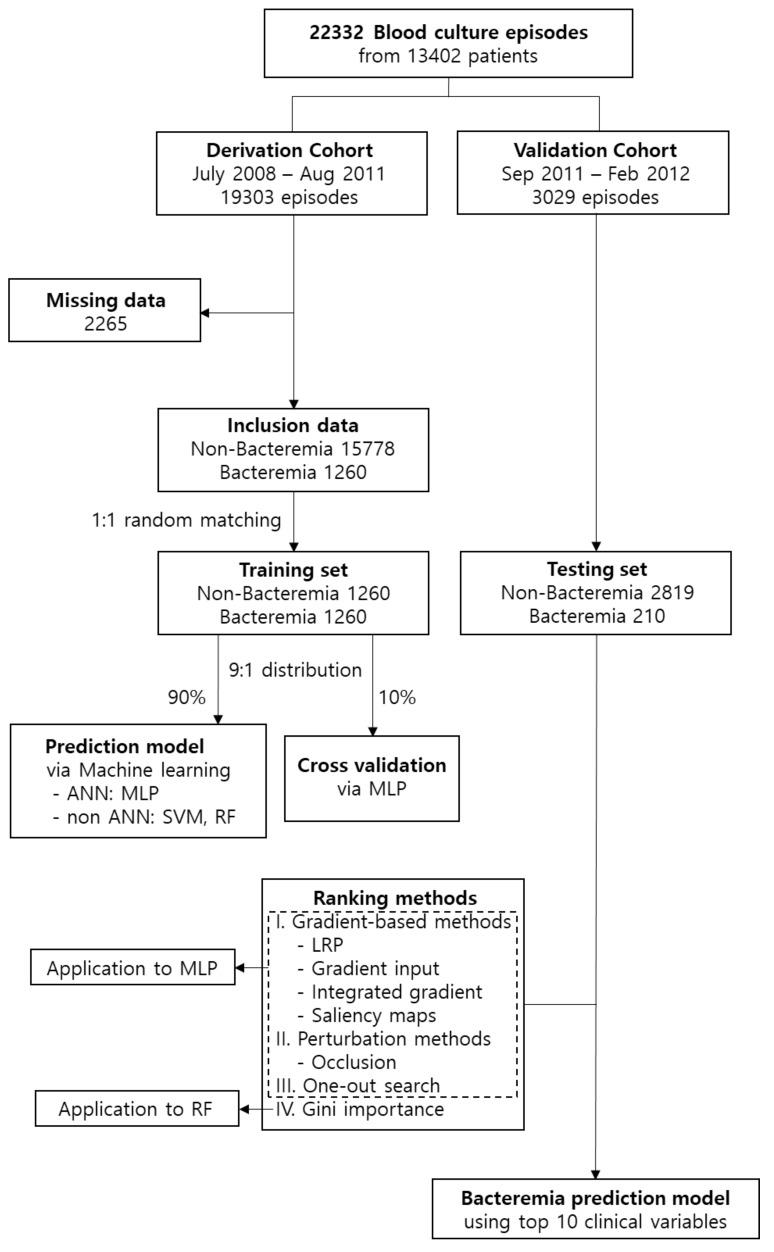
Flow chart of the study design and analysis. ANN: artificial neural network; LRP, layer-wise relevance propagation; MLP, multi-layer perceptron; RF, random forest; and SVM, support vector machine.

A total of 1260 bacteraemia and 15,778 non-bacteraemia episodes from blood cultures were included in the analysis. When training artificial intelligence-based models with imbalanced data with significantly higher negative results than positive results, negative outcomes may be predicted [22]. Thus, the ratio of bacteraemia to non-bacteraemia was adjusted to 1:1 by random matching. We attempted to apply the over-sampling method for positive results using methods such as “Synthetic Sampling with Data Generation”; however, the performance did not improve. Finally, 1260 non-bacteraemia episodes were used for the prediction model (Figure 1). With the distribution of the training set at a 9:1 ratio, 90% of the data were used for training, and 10% of the data were used for internal cross-validation to find the optimized ANN model. Then, additional true validation was performed again with the testing set of 210 bacteraemia episodes and 2819 non-bacteraemia episodes [23,24].

### 2.3. Clinical Variables for Training

We used 20 clinical variables as predictors of bacteraemia (Table 1). Each variable is a known significant clinical factor and was related to bacteraemia in previous prediction models [19,20,25,26]. This study is a retrospective analysis of the medical records; therefore, we included only clinical data that could be extracted as objective numerical values and we excluded symptoms and signs that could be subjective and inaccurate. The model herein differs from the Bayesian model. In the Bayesian model, each variable is stratified and divided into sections and then analysed with respect to bacteraemia risk [18]; however, in this study, the model was trained by continuous numbers without stratification. Information on the intensive care unit (ICU) treatment of the patients and insertion of catheters was related to the time of the blood culture. The results, including antibiotic use up to three weeks prior to the blood culture, steroid use up to two weeks prior to the blood culture, and all vital sign and laboratory data in the last 72 h before the blood culture were measured. Exceptionally, serum albumin and alkaline phosphatase were measured within a week prior to the blood culture tests because they were not measured frequently. As for body temperature and white blood cell count, maximal and minimum body temperatures and clinical number of leucocytosis or leukopenia in the 72 h before the blood culture were considered. The expression “hospital day to blood culture” indicates the period from hospitalization to the blood culture test in number of days. 

### 2.4. Statistical Analysis

The training data were analyzed to develop a prediction model using the conventional statistical approach and machine learning techniques. They included ANN and non-ANN techniques; support vector machine (SVM), and random forest (RF) [3,27]. The multi-layer perceptron (MLP), a representative ANN model, was applied to the learn prediction model of bacteraemia. An ANN is a model that applies the brain’s neural network delivery system as an equation. As a regularization technique, a 0.5 probability drop-out method was applied in the ANN model in this study [28]. Xavier initialization was used to initialize all weights [29]. The mini-batch size was 64, the optimizer was Root Mean Square Propagation [30], the learning rate was 0.001, and the momentum was 0.9. Finally, the early stopping technique was applied to avoid overfitting the performance of the model to the training data [31]. The MLP consists of three layers: input layer, hidden layer, and output layer. The input and output layers are applied for the final results using input or output signals, respectively. The hidden layer is responsible for enhancing the predictive performance of the model through additional nonlinear computation. We applied nodes (128 and 256 in total) of the hidden layer to the MLP and denoted these models as MLP (128) and MLP (256), respectively. The training was conducted for both MLP (128) and MLP (256), and their performances were compared. In addition, the performances of these models were verified by comparison with non-neural network models, namely, SVM and RF. The prediction performance was assessed on the basis of the area under the receiver operating characteristic curve (AUC), sensitivity, and specificity from the validation data.

Furthermore, we used a perturbation-based method and a gradient-based method to demonstrate the validity of the analysis process and the result derivation. Perturbation (occlusion) is a technique to determine the influence of a feature by observing the changing trend of the output feature when the input value is slightly changed [32]. The gradient-based method consists of layer-wise relevance propagation, gradient input, integrated gradients, and saliency maps. It is a technique that determinies the influence of features on the basis of the gradient of the input feature with respect to the output value of the model [33,34,35,36]. The one-out search (OOS) is a technique that learns a model by sequentially excluding feature variables one by one in a model learning process in which a large number of feature variables are input. The difference in performance is then calculated, and the ranking of variables is set in an order based on the performance change. The Gini importance is one of the most important methods of feature selection for measuring the influence characteristics of variables in RF analysis techniques. It is used for comparison with non-ANN techniques [37]. Thus, the rankings of the characteristics for each sample were summed and sorted in an ascending order based on the aforementioned methods.

### 2.5. Ethics Approval and Consent to Participate

The protocol for this retrospective study was reviewed and approved by the Institutional Review Board of Gangnam Severance Hospital, Yonsei University College of Medicine in Seoul, Korea (Reg. No. 3-2018-0165). The board waived the requirement for informed consent. All procedures were conducted in accordance with the guidelines of the Declaration of Helsinki. 

## 3. Results

### 3.1. Bacteraemia and Non-Bacteraemia Groups

A total of 1260 true bacteraemia and 1260 non-bacteraemia episodes were identified from 13,402 patients. Baseline demographic features and comparison of clinical variables between the bacteraemia and the non-bacteraemia groups are presented in Table 1. Significant differences in all 20 clinical variables were observed between the two groups. In particular, the onset age was higher in the bacteraemia group (bacteraemia vs non-bacteraemia group: 64.4 vs 60.1 years, *p* < 0.001), the systolic blood pressure was lower (91.6 vs 99.4 mmHg, *p* < 0.001), the maximum body temperature (38.1 vs 37.6 °C, *p* < 0.001) and the heart rate (115.7 vs 105.7 beats/min, *p* < 0.001) were higher in the bacteraemia group, as expected. Furthermore, in the bacteraemia group, serum C-reactive protein (CRP), alkaline phosphatase, and white blood cell count were significantly higher than in the non-bacteraemia group, and serum albumin concentration and platelet count were lower (*p* < 0.001). The prothrombin time was prolonged, and the duration of hospitalization (24.6 vs 10.3 days, *p* < 0.001), ICU care (32.6 vs 18.7%, *p* < 0.001), central venous catheter placement (9.1 vs 0%, *p* < 0.001), and steroid use (37.9 vs 34.0%, *p* = 0.045) or antibiotics use (46.9% vs 41.7%, *p* = 0.009) in the bacteraemia group were significantly higher than in the non-bacteraemia group.

### 3.2. Bacteraemia Prediction with Various Machine Learning Techniques

It was found that there were clinically significant differences in the examined variables between the bacteraemia and the non-bacteraemia groups; therefore, we attempted to develop a model to predict bacteraemia by repeated learning and precise validation. The aforementioned 20 clinical variables were analyzed to develop a prediction model using the MLP, SVM, and RF techniques (Table 2).

**Figure 2:**
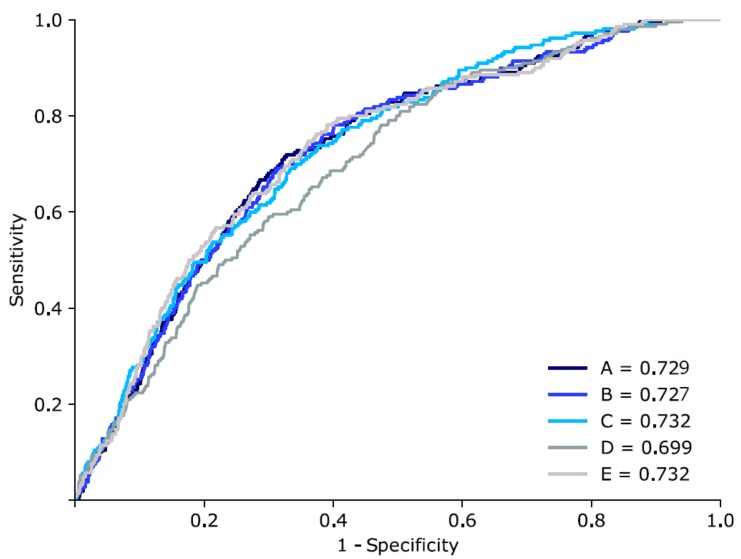
Area under the receiver operating characteristic curve of the bacteremia predictions. A: MLP model with 128 nodes of the hidden layer, B: MLP model with 256 nodes of the hidden layer, C: RF model, D: support vector machine model, and E: combination model of the MLP and RF.

The prediction performance for bacteraemia of the SVM model was the lowest (AUC; 0.699, 95% confidence intervals [CIs]; 0.687–0.700), even though the AUC was 0.7 in the conventional Bayesian statistical method. The highest prediction performance was found for the RF model (AUC; 0.732 [95% CI; 0.722–0.733]). Additionally, the model in which RF and MLP were combined with 128 and 256 nodes of the hidden layer also exhibited high prediction performance (AUC; 0.732 [95% CI; 0.728–0.735]). In MLP (128), the AUC was 0.729 (95% CI; 0.712–0.728) and in MLP (256), the AUC was 0.727 (95% CI; 0.713–0.727). Comparing the sensitivity, the two MLP models showed the highest sensitivity (0.810), whereas the RF model showed the lowest sensitivity (0.682) (Table 2, Figure 2).

### 3.3. Methods for the Ranking of the Variables

To explain the machine learning analysis process, we used various techniques to rank the influence of the variables: four gradient-based methods, one perturbation method, and OOS were used in the MLP, the most remarkable machine learning technique in our study. The results of the three techniques (layer-wise relevance propagation, gradient input, and integrated gradients) are similar; therefore, only the layer-wise relevance propagation results are typically presented. For comparison of the results, we listed the rankings using the Gini importance method used in RF and confirmed that these rankings were applicable to other machine learning methods. Table 3 summarizes the ranking of the OOS and the Gini importance methods, and Supplementary Table S1 shows other rankings. In the OOS method, serum alkaline phosphatase and platelet count were the most and second-most influential variables, respectively. When the influence of each variable was analyzed using the Gini importance method, age and prothrombin time were the most and second-most influential variables, respectively. The alkaline phosphatase and the platelet count, which had a strong influence and ranked high in OOS, still ranked within the top 10 by Gini importance.

### 3.4. Validation by Addition or Subtraction of the Ranking Order

**Figure 3:**
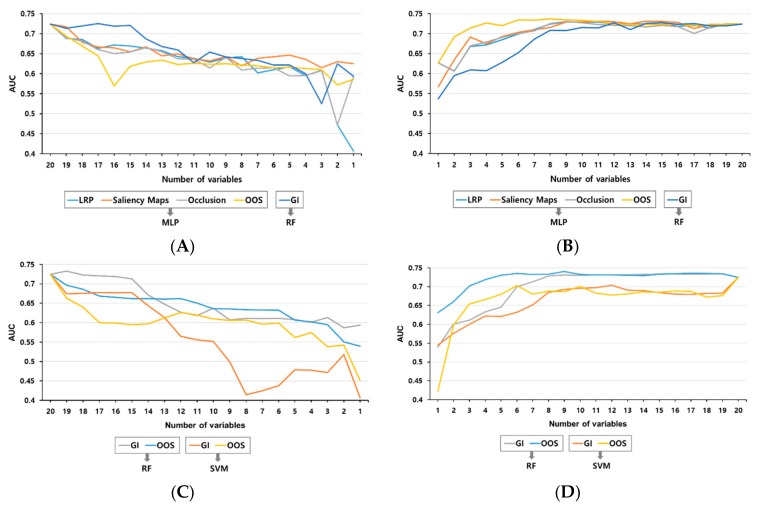
Changing trend of the area under the receiver operating characteristic curve for variables added or removed by ranking. (**A**) Analysis with removed variables according to ranking. (**B**) Analysis with added variables according to ranking. (**C**) Non-ANN analysis with removed variables according to ranking. (**D**) Non-ANN analysis with added variables according to ranking. GI, Gini importance; LRP, Layer-wise Relevance Propagation; OOS, one-out search. ANN, artificial neural network; MLP, multi-layer perceptron; RF, random forest; and SVM, support vector machine.

Regarding the explaining methods, the features of the upper rank were removed from the order, and the prediction models were trained using MLP. Then the upper rank was added in the order again, and the validation was performed after training with MLP. Only the ranking of the Gini importance method was trained and verified with RF. When the variables were removed one by one, the AUC slope of the predictive model fell most sharply with the OOS method, suggesting that the variables were influential. When the variables were added one by one in order using the OOS method, the AUC of the predictive model was also the highest. After the 10th variable, the AUC was not affected either with the addition or with the subtraction models. According to these results, OOS is the best model for measuring the influence of clinical variables (Figure 3A,B).

In order to compare the influence of the variables based on the ranking calculated by OOS with the machine learning algorithms, RF and SVM were applied instead of MLP. Furthermore, ranking by Gini importance methods was applied in the machine learning algorithm for comparison. After examining the performance change, it was found that the ranking of the variables calculated by MLP had the same effect in other non-ANN machine learning algorithms (Figure 3C,D). The AUC of RF and SVM were verified, revealing a slight slope difference for the two method. In general, the addition of up to top 10 variables also influenced the AUC of bacteraemia prediction. The addition of variables except the 10 most influential ones had no significant impact on bacteraemia prediction. Especially when using OOS, the slope width was the largest.

## 4. Discussion

The classic models for predicting prognosis in acute infectious disease are based on the Acute Physiology and Chronic Health Evaluation score using logistic regression [38] or the Sequential Organ Failure Assessment score [39]. However, these approaches are limited to ICU patients or to cases of severe sepsis. These approaches cannot be applied easily to patients who are not in clinically severe septic shock. Many studies predicting bacteraemia risk using existing statistical models were limited to specific bacterial strains or infectious situation [40,41,42]. The patient’s condition can always deteriorate at an unpredictable moment, and after a diagnosis of a particular strain infection or disease, progress and prognosis can be determined on the basis of accumulated clinical experience. We aimed to present a model for real-time monitoring of overall bacteraemia predictions in naive patients, independent of specific strains or situations.

Compared to conventional statistical models, the ANN-based prediction model MLP showed better predictive value, with an AUC and sensitivity greater than 0.7 and 0.8, respectively. Thus, ANN analysis using MLP was the most ideal prediction model when AUC and sensitivity were integrated in a machine learning method in this study. Recent deep learning algorithms are referred to as ‘black boxes’ due to their internal complexity [43]. Furthermore, with deep learning algorithms, it is not possible to sufficiently explain the basis of the final results and the validity of the derivation process. In the field of medicine, where accuracy is important, there is a growing interest in the process of examining the validity of results generated from artificial intelligence systems to ensure fairness, reliability, and accuracy. 

Consequently, ranking methods were applied to study how the influence of the input variables. Based on the ranking of variables calculated from various algorithms, we confirmed a performance change in the MLP model, and the most accurate ranking method was OOS (Figure 3A,B). We were able to develop a bacteraemia prediction model using MLP with the top 10 variables out of 20 variables. Although other studies have developed similar predictive models for bacteraemia using recent artificial intelligence techniques and have shown significant specificity and sensitivity demonstrating the superiority of artificial intelligence analysis, the novelty of our study is that we compared various artificial intelligence techniques and used the ranking method of analytic variables. We used a ranking scoring system to determine the risk of progression to bacteraemia, suggesting a precise predictable basis. Also, OOS is a meta-algorithm that checks the explanatory power of clinical variables through the performance of a trained model for each subset of functional variables (excluded specific variables), so it has no limit of application, regardless of the deep or shallow network structure. The OOS is not a new interpretable technique but it is more accurate than the other analytic methods of decision tree (Gini importance) that were widely used in previous studies.

In this study, we also identified serum alkaline phosphatase as one of the most influential variables. The elevation of alkaline phosphatase often indicates hepatobiliary or bones problems. They may also indicate malnutrition, malignancy, or uncommonly serious infection. Testing alkaline phosphatase has several advantages, even if it is not usually considered a significant predictor of infection or bacteraemia. Several studies showed the elevation of alkaline phosphatase count in patients with bacteraemia [44,45]. Alkaline phosphatase levels can be determined relatively fast (usually in an hour). Additionally, its analysis is cheap and included in regular liver function tests or chemistry laboratory tests [46]. However, the relation between the presence of infectious organisms and alkaline phosphatase elevation and disease in a patient must be studied to determine if alkaline phosphatase is a powerful predictor.

There are some limitations in this study. (1) This study is an analysis of data from a single center and requires verification through external validation. (2) As a retrospective study, there are limitations in using variables. We included 20 other variables that could be quantified comparatively objectively; however, other important clinical symptoms and signs that cannot be obtained with electronic medical records were not included. (3) The 24 h period, during which bacteraemia episodes were considered as duplicates, can provide a large amount of data; however, the clinical information regarding blood culture episodes may be weighted in patients who had followed up cultures at 3–4 day intervals. (4) “Big data analysis using artificial intelligence has a difficulty in extracting vast amount of medical information”. Therefore, such an analysis might miss recently updated clinical information. We could not add the current medical information and we used only the medical information extracted previously for comparison with conventional statistical analyses. However, the use of the same dataset may be an advantage as the results can be directly compared with those of obtained using existing statistical techniques. (5) Finally, ranking methods were used to better explain the results of artificial intelligence analysis; however, there exists uncertainty regarding the interpretation of results through machine learning techniques based on ranking methods. The best solution would be to apply the prediction model created with the top 10 ranked variables to other multi-centers to verify the predictability and to study the cut-off values in interpreting the predicted score.

If we apply a predictive model made with MLP to real clinical practice, we can use a clinical scoring system to predict true bacteraemia after a blood culture test in real-time. This may improve the patient prognosis, as it could suggest the immediate use of antibiotics in patients with bacteraemia or septic shock and prevent unnecessary use/abuse of antibiotics. Ultimately, the bacteraemia prediction model will lead to the control of antibiotic use and inhibition of antibiotic resistance, allowing better prognoses with timely interventions. To improve the performance of the prediction, a larger amount of clinical data and external validation are necessary. In addition, the patients’ clinical information from the actual electronic medical record should be linked directly to the prediction model, and a system to check the predicted score of bacteraemia in real-time should be developed.

Artificial intelligence analysis has been used as a repetitive deep learning technique mainly using images in the existing medical environment. In other fields, Artificial intelligence is deeply involved in our lives also in other fields, including personalized advertising, autonomous navigation, and language translation. Continuous extraction of data from electronic medical records and real-time calculation may allow physicians to identify sepsis and septic shock progression earlier and provide timely interventions to reduce mortality and morbidity [5,12,13]. We are convinced that this can also greatly change the quality of our lives. 

## 5. Conclusions

In conclusion, the bacteraemia prediction model using ANN, especially MLP, is not inferior to the conventional statistical approaches. The novelty of this study is the use of the ranking method of clinical variables, showing that the OOS was the most influential ranking method. With the top 10 clinical variables, including one of the most influential variables, i.e., serum alkaline phosphatase, it is possible to develop a scoring system and real-time monitoring to predict bacteraemia, which will help clinicians’ rapid and accurate diagnosis.

## Figures and Tables

**Table 1:** Comparison of the clinical characteristics of two groups classified according to bacteremia.

	Bacteremia, Yes (*n* = 1260)	Bacteremia, No (*n* = 1260)	*p*-Value
Age, years	64.4 ± 15.3	60.1 ± 17.3	<0.001
Sex, male	558 (44.3%)	533 (42.3%)	0.306
Vital sign			
SBP, mmHg	91.6 ± 23.2	99.4 ± 22.8	<0.001
Body temperature (min), °C	36.1 ± 0.4	36.1 ± 0.3	0.578
Body temperature (max), °C	38.1 ± 0.9	37.6 ± 0.9	<0.001
Heart rate, beats/min	115.7 ± 29.2	105.7 ± 28.5	<0.001
Respiratory rate, /min	25.5 ± 8.6	23.6 ± 8.9	<0.001
Laboratory data			
Creatinine, mg/dL	1.6 ± 1.5	1.2 ± 1.3	0.317
Albumin, g/dL	2.8 ± 0.6	3.2 ± 0.7	<0.001
CRP, mg/L	1.6 ± 1.5	1.2 ± 1.3	<0.001
Alkaline phosphatase, IU/L	191.3 ± 213.7	136.0 ± 141.0	<0.001
WBC count (min), /μL	10.3 ± 6.2	9.3 ± 5.5	<0.001
WBC count (max), /μL	14.8 ± 8.9	11.9 ± 7.3	<0.001
Platelet count, /μL	185.9 ± 134.8	218.7 ± 129.8	<0.001
Prothrombin time, s	18.5 ± 8.9	15.9 ± 6.6	<0.001
Clinical information			
Hospital day to blood culture, days	24.6 ± 75.3	10.3 ± 17.6	<0.001
ICU care, yes	410 (32.6%)	235 (18.7%)	<0.001
Central venous catheter, yes	114 (9.1%)	0 (0%)	<0.001
Steroid therapy, yes	477 (37.9)	429 (34.0)	0.045
Antibiotic therapy, yes	591 (46.9%)	526 (41.7%)	0.009

Data are expressed as mean ± standard deviation or n (%). Abbreviations: SBP, systolic blood pressure; DBP, diastolic blood pressure; CRP, C-reactive protein; WBC, white blood cell; and ICU, intensive care unit.

**Table 2:** Results and performance of the bacteremia predictions using various machine learning techniques.

Model	Algorithms	AUC (95% CI)	Sensitivity	Specificity
Previous	Naive Bayesian	0.7	-	-
A	MLP (128)	0.729 (0.712–0.728)	0.810	0.589
B	MLP (256)	0.727 (0.713–0.727)	0.810	0.532
C	RF	0.732 (0.722–0.733)	0.682	0.655
D	SVM	0.699 (0.687–0.700)	0.692	0.553
E	MLP (128) + MLP (256) + RF	0.732 (0.728–0.735)	0.793	0.571

Standard errors and 95% confidences were obtained from 10-fold cross-validation. Abbreviations: AUC, area under the receiver operating characteristic curve; SE, standard error; CI, confidence interval.

**Table 3:** Influence of the ranking of the clinical variables on bacteremia predictions.

Rank	One-Out Search	Gini Importance
1	ALP	Age
2	Platelet	Prothrombin time
3	Body temperature (max)	Heart rate
4	Systolic BP	Hospital day to blood culture
5	WBC count (min)	Albumin
6	ICU stay	ALP
7	CRP	Platelet
8	Central venous catheter	Body temperature (max)
9	Prothrombin time	WBC count (max)
10	Albumin	Creatinine
11	Steroid use	systolic BP
12	Sex	WBC count (min)
13	Antibiotic	Respiratory rate
14	Body temperature (min)	ICU stay
15	Hospital day to blood culture	Steroid
16	WBC count (max)	Sex
17	Heart rate	Body temperature (min)
18	Respiratory rate	Antibiotics
19	Age	CRP
20	Creatinine	Central venous catheter

Abbreviations: ALP, alkaline phosphatase; BP, blood pressure; CRP, C-reactive protein.

## Supplementary Materials

The following are available online at https://www.mdpi.com/2077-0383/8/10/1592/s1, Table S1: Influence of the ranking of clinical variables on bacteremia predictions.
