# Supplementary material for: Early Detection of Bacteraemia Using Ten Clinical Variables with an Artificial Neural Network Approach
Source: J Clin Med. 2019 Oct 2;8(10):1592. doi: 10.3390/jcm8101592 (PMC6832527; doi:10.3390/jcm8101592)
Supplement: Supplementary file 1 [file jcm-08-01592-s001.pdf]

Table S1. Influence of the ranking of clinical variables on bacteremia predictions.

Rank	Gradient-based methods				Perturbation based methods
	Layer-wise Relevance Propagation	Gradient Input	Integrated Gradients	Saliency Maps	Occlusion
1	ALP	ALP	ALP	Hospital day	ALP
2	Age	Age	Age	Platelet	Age
3	Platelet	Platelet	Platelet	ALP	Platelet
4	Hospital day	Hospital day	Hospital day	Age	Hospital day
5	Heart rate	CRP	Heart rate	ICU stay	CRP
6	CRP	Heart rate	CRP	Heart rate	Heart rate
7	Systolic BP	Systolic BP	systolic BP	Systolic BP	Systolic BP
8	Body temperature (max)	Body temperature (max)	Body temperature (max)	CRP	Body temperature (max)
9	WBC count (min)	WBC count (min)	WBC count (min)	Body temperature (max)	WBC count (min)
10	Creatinine	Creatinine	Creatinine	Steroid use	Creatinine
11	Body temperature (min)	WBC count (max)	Body temperature (min)	WBC count (min)	WBC count (max)
12	WBC count (max)	Respiratory rate	WBC count (max)	Creatinine	Albumin
13	Albumin	Albumin	Albumin	Antibiotic	Body temperature (min)
14	Prothrombin time	Body temperature (min)	Prothrombin time	Sex	Respiratory rate
15	Steroid use	Prothrombin time	Respiratory rate	Central venous catheter	Prothrombin time
16	Respiratory rate	Steroid use	Steroid use	WBC count (max)	Steroid use
17	Antibiotic	Antibiotic	Antibiotic	Respiratory rate	Antibiotic
18	ICU stay	Sex	ICU stay	Body temperature (min)	ICU stay
19	Sex	ICU stay	Sex	Albumin	Sex

20	Central venous catheter	Central venous catheter	Central venous catheter	Prothrombin time	Central venous catheter

Abbreviations: ALP, alkaline phosphatase; BP, blood pressure; CRP, c-reactive protein; ICU, intensive care unit; and WBC, white blood cell.
